# Influenza and Tdap Vaccination Coverage Among Pregnant Women — United States, April 2018

**DOI:** 10.15585/mmwr.mm6738a3

**Published:** 2018-09-28

**Authors:** Katherine E. Kahn, Carla L. Black, Helen Ding, Walter W. Williams, Peng-Jun Lu, Amy Parker Fiebelkorn, Fiona Havers, Denise V. D’Angelo, Sarah Ball, Rebecca V. Fink, Rebecca Devlin

**Affiliations:** ^1^Leidos, Reston, Virginia; ^2^Immunization Services Division, National Center for Immunization and Respiratory Diseases, CDC; ^3^CFD Research Corporation, Huntsville, Alabama; ^4^Division of Bacterial Diseases, National Center for Immunization and Respiratory Diseases, CDC; ^5^Division of Reproductive Health, National Center for Chronic Disease Prevention and Health Promotion, CDC; ^6^Abt Associates, Inc., Cambridge, Massachusetts.

Vaccinating pregnant women with influenza and tetanus toxoid, reduced diphtheria toxoid, and acellular pertussis (Tdap) vaccines can reduce the risk for influenza and pertussis for themselves and their infants. The Advisory Committee on Immunization Practices (ACIP) recommends that all women who are or might be pregnant during the influenza season receive influenza vaccine, which can be administered any time during pregnancy (*1*). The ACIP also recommends that women receive Tdap during each pregnancy, preferably from 27 through 36 weeks’ gestation (*2*). To assess influenza and Tdap vaccination coverage among women pregnant during the 2017–18 influenza season, CDC analyzed data from an Internet panel survey conducted during March 28–April 10, 2018. Among 1,771 survey respondents pregnant during the peak influenza vaccination period (October 2017–January 2018), 49.1% reported receiving influenza vaccine before or during their pregnancy. Among 700 respondents who had a live birth, 54.4% reported receiving Tdap during their pregnancy. Women who reported receiving a provider offer of vaccination had higher vaccination coverage than did women who received a recommendation but no offer and women who did not receive a recommendation. Reasons for nonvaccination included concern about effectiveness of the influenza vaccine and lack of knowledge regarding the need for Tdap vaccination during every pregnancy. Provider offers or referrals for vaccination in combination with patient education could reduce missed opportunities for vaccination and increase vaccination coverage among pregnant women.

An Internet panel* survey was conducted to assess end-of-season influenza vaccination coverage and Tdap coverage estimates among women pregnant during the 2017–18 influenza season, as previously described (*3*,*4*). The survey was conducted during March 28–April 10, 2018, among women aged 18–49 years who reported being pregnant at any time since August 1, 2017, through the date of the survey. Among 14,858 women who entered the survey site, 2,342 reported they were eligible, and of these, 2,236 completed the survey (cooperation rate = 95.5%).^†^ Data were weighted to reflect the age, race/ethnicity, and geographic distribution of the total U.S. population of pregnant women. Analysis of influenza vaccination coverage was limited to 1,771 women who reported being pregnant any time during the peak influenza vaccination period (October 2017–January 2018). A woman was considered to have been vaccinated against influenza if she reported receiving a dose of influenza vaccine (before or during her most recent pregnancy) since July 1, 2017. To accommodate the optimal timing for Tdap vaccination during 27 through 36 weeks’ gestation, analysis of Tdap coverage was limited to women who reported being pregnant any time since August 1, 2017, and who had a live birth. A woman was considered to have received Tdap if she reported receiving a dose of Tdap vaccine during her most recent pregnancy. Among 815 women who had a live birth, 115 (14.1%) were excluded from analysis because they did not know if they had ever received Tdap vaccination (11.4%) or did not know if the Tdap vaccine was received during their pregnancy (2.7%), leaving a final analytic sample of 700. An estimate of the proportion of pregnant women who received both recommended maternal vaccines was assessed among these 700 women. A difference was noted as an increase or decrease when there was a ≥5 percentage-point difference between any values being compared.^§^

Among pregnant women, 49.1% reported receiving a dose of influenza vaccine since July 1, 2017 (Table); Tdap coverage during pregnancy was 54.4% among women with a recent live birth. Receipt of both influenza and Tdap vaccines (i.e., being fully vaccinated) was reported by 32.8% of women with a recent live birth (Figure 1). Influenza vaccination coverage increased with increasing number of provider visits since July 1, 2017, ranging from 18.1% (0 visits) to 56.8% (>10 visits) (Table).

**TABLE:** Influenza and tetanus toxoid, reduced diphtheria toxoid, and acellular pertussis vaccination (Tdap) coverage among pregnant women, by selected characteristics — Internet panel survey, United States, April 2018

Characteristic	Influenza*		Tdap^†^	
	No. (weighted %)	Vaccinated, weighted %	No. (weighted %)	Vaccinated, weighted %
**Total**	**1,771 (100.0)**	**49.1**	**700 (100.0)**	**54.4**
Vaccinated before pregnancy	213 (—)	12.3	N/A	N/A
Vaccinated during pregnancy	681 (—)	36.8	396 (—)	54.4
**Age group (yrs)**				
18–24	345 (25.3)	42.7^§^	126 (24.1)	49.0
25–34	1,064 (55.4)	50.5	444 (57.5)	57.9^§^
35–49^¶^	362 (19.3)	53.4	130 (18.4)	50.6
Race/Ethnicity**				
White, non-Hispanic^¶^	1,167 (50.4)	52.5	502 (57.3)	59.3
Black, non-Hispanic	192 (18.9)	35.6^§^	65 (16.6)	42.9^§^
Hispanic	270 (23.6)	51.3	78 (18.7)	48.8^§^
Other, non-Hispanic	142 (7.1)	53.0	55 (7.4)	56.5
**Education**				
≤High school diploma	385 (24.2)	41.8^§^	145 (22.7)	46.2^§^
Some college, no degree	429 (24.9)	40.0^§^	192 (28.1)	54.5
College degree	704 (37.9)	56.0	274 (37.3)	57.8
>College degree^¶^	253 (12.9)	59.7	89 (11.9)	59.0
**Marital status**				
Married^¶^	1,101 (56.7)	56.9	471 (62.7)	58.6
Unmarried	670 (43.3)	38.8^§^	229 (37.3)	47.4^§^
**Insurance coverage^††^**				
Private/Military only^¶^	939 (50.1)	55.3	369 (50.0)	58.8
Any public	752 (44.9)	44.2^§^	314 (47.3)	50.8^§^
No insurance	80 (5.0)	30.1^§^	<30(—^§§^)	—^§§^
**Employment status^¶¶^**				
Working^¶^	959 (53.7)	53.5	330 (46.8)	52.9
Not working	812 (46.3)	43.9^§^	370 (53.2)	55.8
Poverty status***				
At or above poverty^¶^	1,416 (77.3)	52.0	538 (73.5)	58.3
Below poverty	352 (22.7)	38.8^§^	162 (26.5)	43.7^§^
**High-risk condition^†††^**				
Yes^¶^	651 (42.5)	54.0	N/A	N/A
No	887 (57.5)	46.3^§^	N/A	N/A
**No. of provider visits since July 2017**				
None	30 (1.8)	18.1^§^	N/A	N/A
1–5	385 (22.3)	37.4^§^	N/A	N/A
6–10	677 (38.8)	49.9^§^	N/A	N/A
>10^¶^	679 (37.0)	56.8	N/A	N/A
**Provider vaccination recommendation/offer^§§§^**				
Offered^¶^	1,189 (66.6)	63.8	489 (67.4)	73.5
Recommended with no offer	244 (14.5)	37.6^§^	78 (11.9)	38.3^§^
Recommended with no offer, referral received	108 (6.1)	47.9^§^	39 (6.3)	56.1^§^
Recommended with no offer, no referral received	136 (8.4)	30.1^§^	39 (5.7)	18.5^§^
No recommendation	308 (19.0)	9.0^§^	133 (20.7)	1.6^§^

**Abbreviation:** N/A = not applicable.

* Women pregnant any time during October–January were included in the analysis to assess influenza vaccination coverage for the 2017–18 season. Women who received an influenza vaccination since July 1, 2017, before or during their pregnancy were considered vaccinated.

^†^ Women pregnant any time since August 1, 2017, and had a live birth were included in the analysis to assess Tdap coverage. Women who received a Tdap vaccination during their recent pregnancy were considered vaccinated.

^§^ ≥5 percentage-point difference compared with reference group.

^¶^ Reference group for comparison within subgroups.

** Race/ethnicity was self-reported. Women identified as Hispanic might be of any race. Women categorized as white, black, or other race were identified as non-Hispanic. The “other” race category included Asians, American Indians or Alaska Natives, Native Hawaiians or other Pacific Islanders, and women who selected “other” or multiple races.

^††^ Women considered to have any public insurance selected at least one of the following when asked what kind of medical insurance they had: Medicaid, Medicare, Indian Health Service, state sponsored medical plan, or other government plan. Women considered to have private/military insurance selected private medical insurance and/or military medical insurance and did not select any type of public insurance.

^§§^ Estimates not reported because sample size was <30.

^¶¶^ Women who were employed for wages and self-employed were categorized as working; those who were out of work, homemakers, students, retired, or unable to work were categorized as not working.

*** Poverty status was defined based on the reported number of people and children living in the household and annual household income, according to the U.S. Census poverty thresholds (https://www.census.gov/data/tables/time-series/demo/income-poverty/historical-poverty-thresholds.html).

^†††^ Conditions associated with increased risk for serious medical complication from influenza, including chronic asthma, a lung condition other than asthma, a heart condition, diabetes, a kidney condition, a liver condition, obesity, or a weakened immune system caused by a chronic illness or by medicines taken for a chronic illness. Women who were missing information were not included in the analysis for high-risk conditions (n = 233).

^§§§^ Excluded women who did not report having a provider visit since July 2017 (n = 30) for the influenza vaccination coverage analysis; no women were excluded for the Tdap vaccination coverage analysis.

**FIGURE 1:**
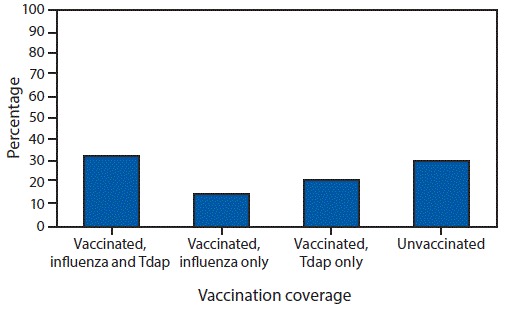
Tetanus toxoid, reduced diphtheria toxoid, and acellular pertussis (Tdap) and influenza vaccination coverage* among women with a recent live birth — Internet panel survey, United States, April 2018 * Weighted percentage of women who reported 1) receiving influenza vaccine before or during pregnancy since July 1, 2017, and receiving Tdap vaccine during most recent pregnancy; 2) receiving influenza vaccine before or during pregnancy since July 1, 2017, but not receiving Tdap vaccine during most recent pregnancy; 3) receiving Tdap vaccine during most recent pregnancy but not receiving influenza vaccine before or during pregnancy since July 1, 2017; or 4) not receiving influenza vaccine before or during pregnancy since July 1, 2017, and not receiving Tdap vaccine during most recent pregnancy.

Among women pregnant any time during October 2017–January 2018, 66.6% reported receiving a provider offer of influenza vaccination, 14.5% received a recommendation but no offer, and 19.0% received no recommendation (Table). The percentages of women in these groups who received influenza vaccine were 63.8%, 37.6%, and 9.0%, respectively. Among women who reported that their provider recommended but did not offer influenza vaccination, 42.1% received a referral^¶^ to get vaccinated elsewhere. Women with a referral were more likely to receive an influenza vaccination (47.9%) than were women who received a provider recommendation but did not receive a referral (30.1%).

Among women with a live birth since August 1, 2017, 67.4% reported receiving a provider offer of Tdap, 11.9% received a recommendation but no offer, and 20.7% received no recommendation (Table). The percentages of these women who received Tdap among these groups were 73.5%, 38.3%, and 1.6%, respectively. Among women who reported that their provider recommended but did not offer Tdap, 52.9% received a referral.^¶^ Among women who received a referral, 56.1% received Tdap, compared with 18.5% of women who received a provider recommendation but did not receive a referral.

The most commonly reported main reason for not receiving influenza vaccination before or during pregnancy was belief that the vaccine is not effective (20.2%) (Figure 2). The most common main reason for not receiving Tdap during pregnancy was a lack of knowledge about the need to be vaccinated during every pregnancy (45.1%): 31.6% of women who did not receive vaccine during pregnancy reported having been vaccinated previously, and 13.5% reported not knowing they were supposed to receive Tdap during their recent pregnancy. The second most commonly reported main reason for nonreceipt of both vaccines was concern about safety risks to the baby (16.0% and 13.5% of women who did not receive influenza vaccine or Tdap, respectively).

**FIGURE 2:**
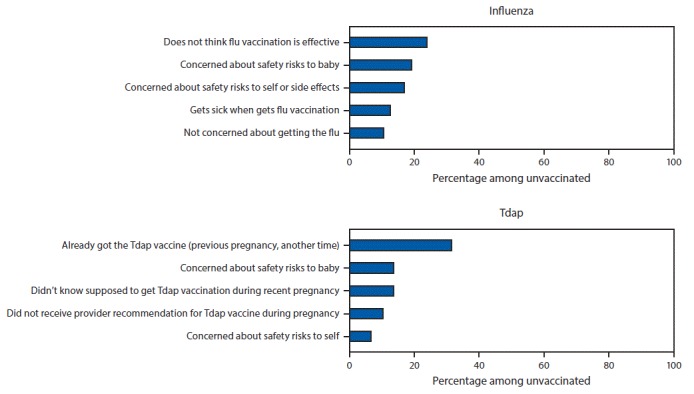
Main reasons for not receiving influenza vaccine* or tetanus toxoid, reduced diphtheria toxoid, and acellular pertussis vaccine (Tdap)^†^ among pregnant women who did not receive influenza vaccine (n = 817) or Tdap (n = 297) — Internet panel survey, United States, April 2018 * Main reason for not receiving influenza vaccination among women pregnant any time during October–January in the 2017–18 influenza season who were not vaccinated as of early April 2018 (n = 817). Excluded women who were not vaccinated but did not provide information on the reason for not being vaccinated (n = 1). ^†^ Main reason for not receiving Tdap among women who were recently pregnant at the time of the survey (March 28–April 10, 2018), had a live birth, and were not vaccinated during their most recent pregnancy (n = 297). Excluded women who were not vaccinated but did not provide information on the reason for not being vaccinated (n = 7).

## Discussion

Findings from this survey indicate that many pregnant women are unvaccinated, and they and their babies continue to be vulnerable to influenza and pertussis infection and potentially serious complications including hospitalization and death. Providers are encouraged to strongly recommend vaccines that their patients need and either administer needed vaccines or refer patients to a vaccination provider (*5*). Vaccination coverage, regardless of vaccine type, was highest among pregnant women with a provider offer of vaccination, which has been reported previously (*4*,*6*). For providers unable to offer vaccination, referring patients to a vaccination provider was also shown to help improve vaccination coverage, especially for Tdap.

Missed opportunities to vaccinate were common, even among women with multiple health care visits. Many pregnant women reported not receiving a provider recommendation for vaccination, which might be partly attributable to differences in perception of a provider recommendation between patients and providers. Results from a survey of obstetric care providers conducted by the American College of Obstetricians and Gynecologists (ACOG) suggest that whereas providers believe they are giving a recommendation for vaccination, the recommendation might not be strong enough to be remembered by patients (*7*). CDC has resources to assist providers in effectively communicating the importance of vaccination, such as sharing specific reasons why the recommended vaccine is right for the patient and highlighting positive experiences with vaccines (personal or practice).** Another available resource is the ACOG immunization toolkit which includes communication strategies for providers.^††^ The toolkit also includes extensive information on vaccine financing and coding that could address perceived financial barriers, a commonly reported barrier to stocking vaccine (*8*).

Examination of reasons for nonvaccination provides insight into why some women received influenza vaccination or Tdap, but not both, and further highlights the importance of an effective provider recommendation for vaccination. Provider awareness of concern about effectiveness of the influenza vaccine, lack of knowledge about the recommendation to receive Tdap during every pregnancy, and concern about safety risks to the baby related to both vaccines can help providers address these issues with their patients through education and thus strengthen their recommendations for vaccination.

The findings in this report are subject to at least four limitations of the survey, three of which have been reported previously (*3*,*4*). First, this was a nonprobability sample, and results might not be generalizable to all pregnant women in the United States. Second, vaccination status was self-reported and might be subject to recall bias or social desirability bias. Third, the Tdap coverage estimates might be subject to uncertainty, given the exclusion of 14.1% of women with unknown Tdap vaccination status from estimations of Tdap coverage. Finally, although Internet panel surveys of pregnant women have been conducted since the 2010–11 influenza season, a methodology change increased the proportion of women who were able to complete the 2018 survey on a smartphone or other handheld device and limits the ability to make comparisons to estimates from previous seasons; however, both influenza vaccination and Tdap coverage estimates were similar to those reported from the April 2017 survey (*4*,*6*). Despite these limitations, Internet panel surveys are considered a useful assessment tool for timely evaluation of influenza vaccination and Tdap coverage among pregnant women.

Despite ACIP recommendations, maternal vaccination with influenza and Tdap vaccines is suboptimal, and missed opportunities to vaccinate are common. Findings in this report reinforced the importance of a provider’s recommendation and offer of vaccination, or referral, to pregnant patients in receipt of recommended vaccination. Vaccination coverage of pregnant women can be increased by implementation of evidence-based practices, as indicated by the Standards for Adult Immunization Practices, such as screening patients for recommended vaccinations at every opportunity, reminders to notify providers that their patients need vaccinations, and patient education about ACIP vaccination recommendations and safety and benefits of maternal vaccination (*5*,*9*,*10*).

**Summary**

**What is already known about this topic?**

Vaccinating pregnant women with influenza and tetanus toxoid, reduced diphtheria toxoid, and acellular pertussis (Tdap) vaccines can reduce the risk for severe complications from influenza and pertussis for themselves and their infants.

**What is added by this report?**

During the 2017–18 influenza season, 49.1% of pregnant women received influenza vaccination before or during pregnancy, 54.4% of women with a live birth received Tdap during pregnancy, and 32.8% received both recommended vaccines.

**What are the implications for public health practice?**

Implementing the Standards for Adult Immunization Practice to assess pregnant women’s vaccination status, provide an effective vaccination recommendation, administer vaccines or refer to a vaccination provider for vaccination, and document vaccines administered by providers can help ensure pregnant women are fully vaccinated.

## Footnotes

* https://www.surveysampling.com.

† An opt-in Internet panel survey is a nonprobability sampling survey. The denominator for a response rate calculation cannot be determined because no sampling frame with a selection probability is involved at the recruitment stage. Instead, the survey cooperation rate is provided.

§ Additional information on obstacles to inference in nonprobability samples is available at: https://www.aapor.org/AAPOR_Main/media/MainSiteFiles/NPS_TF_Report_Final_7_revised_FNL_6_22_13.pdf and https://www.aapor.org/getattachment/Education-Resources/For-Researchers/AAPOR_Guidance_Nonprob_Precision_042216.pdf.aspx. Although the estimates reported here have variance, there has been no attempt to quantify the size of the variance.

¶ Referral is defined based on a “yes” response to the question “Did any doctor, nurse, or medical professional suggest that you go someplace else to get the <flu/Tdap> vaccination?”

**
https://www.acog.org/-/media/Departments/Immunization/ImmunizationToolkit.pdf .

††
https://www.cdc.gov/vaccines/hcp/adults/for-practice/standards/recommend.html .

## References

[1] Grohskopf LA, Sokolow LZ, Broder KR, Prevention and control of seasonal influenza with vaccines: recommendations of the Advisory Committee on Immunization Practices—United States, 2017–18 influenza season. MMWR Recomm Rep 2017;66(No. RR-2). 10.15585/mmwr.rr6602a1 28841201 PMC5837399

[2] Liang JL, Tiwari T, Moro P, Prevention of pertussis, tetanus, and diphtheria with vaccines in the United States: recommendations of the Advisory Committee on Immunization Practices (ACIP). MMWR Recomm Rep 2018;67(No. RR-2). 10.15585/mmwr.rr6702a1 29702631 PMC5919600

[3] Ding H, Black CL, Ball S, Influenza vaccination coverage among pregnant women—United States, 2014–15 influenza season. MMWR Morb Mortal Wkly Rep 2015;64:1000–5. 10.15585/mmwr.mm6436a2 26390253

[4] Kahn KE, Black CL, Ding H, Pregnant women and Tdap vaccination, Internet panel survey, United States, April 2017. Atlanta, GA: US Department of Health and Human Services, CDC; 2018. https://www.cdc.gov/vaccines/imz-managers/coverage/adultvaxview/pubs-resources/tdap-report-2017.html

[5] Orenstein WA, Gellin BG, Beigi RH, ; National Vaccine Advisory Committee. Recommendations from the National Vaccine Advisory Committee: standards for adult immunization practice. Public Health Rep 2014;129:115–23. 10.1177/003335491412900203 24587544 PMC3904889

[6] Ding H, Black CL, Ball S, Influenza vaccination coverage among pregnant women—United States, 2016–17 influenza season. MMWR Morb Mortal Wkly Rep 2017;66:1016–22. 10.15585/mmwr.mm6638a2 28957044 PMC5657675

[7] Stark LM, Power ML, Turrentine M, Influenza vaccination among pregnant women: patient beliefs and medical provider practices. Infect Dis Obstet Gynecol 2016;2016:3281975. 10.1155/2016/3281975 27559272 PMC4983379

[8] O’Leary ST, Riley LE, Lindley MC, Immunization practices of U.S. obstetrician/gynecologists for pregnant patients. Am J Prev Med 2018;54:205–13. 10.1016/j.amepre.2017.10.016 29246674 PMC5783738

[9] Community Preventive Services Task Force. The guide to community preventive services. Vaccination. Atlanta, GA: US Department of Health and Human Services, CDC, Community Preventive Services Task Force; 2008. https://www.thecommunityguide.org/topic/vaccination

[10] Mazzoni SE, Brewer SE, Pyrzanowski JL, Effect of a multi-modal intervention on immunization rates in obstetrics and gynecology clinics. Am J Obstet Gynecol 2016;214:617.e1–7. 10.1016/j.ajog.2015.11.018 26627727

